# Phenotypic covariance at species’ borders

**DOI:** 10.1186/1471-2148-13-105

**Published:** 2013-05-28

**Authors:** M Julian Caley, Edward Cripps, Edward T Game

**Affiliations:** 1 Australian Institute of Marine Science, PMB # 3, Townsville MC, Queensland QLD 4810, Australia; 2 School of Mathematics and Statistics, University of Western Australia, Perth, Australia; 3 The Nature Conservancy, Conservation Science, 245 Riverside Drive, West End, Queensland 4101, Australia

## Abstract

**Background:**

Understanding the evolution of species limits is important in ecology, evolution, and conservation biology. Despite its likely importance in the evolution of these limits, little is known about phenotypic covariance in geographically marginal populations, and the degree to which it constrains, or facilitates, responses to selection. We investigated phenotypic covariance in morphological traits at species’ borders by comparing phenotypic covariance matrices (**P**), including the degree of shared structure, the distribution of strengths of pair-wise correlations between traits, the degree of morphological integration of traits, and the ranks of matricies, between central and marginal populations of three species-pairs of coral reef fishes.

**Results:**

Greater structural differences in **P** were observed between populations close to range margins and conspecific populations toward range centres, than between pairs of conspecific populations that were both more centrally located within their ranges. Approximately 80% of all pair-wise trait correlations within populations were greater in the north, but these differences were unrelated to the position of the sampled population with respect to the geographic range of the species.

**Conclusions:**

Neither the degree of morphological integration, nor ranks of **P**, indicated greater evolutionary constraint at range edges. Characteristics of **P** observed here provide no support for constraint contributing to the formation of these species’ borders, but may instead reflect structural change in **P** caused by selection or drift, and their potential to evolve in the future.

## Background

Understanding how and why species’ range limits evolve [1-4], and the extent to which constraints on evolutionary responses are imposed by covariance among traits [5-10] are two important, but currently unresolved, issues in evolutionary biology. The theory of species’ borders evolution reviewed by [4] and evolutionary responses, where multiple co-varying traits are involved [5-10], are both considerably advanced, but empirical tests of these ideas are few [2,4,11]. Moreover, whether responses to selection imposed by multiple co-varying traits constrain evolution at range edges and leads to the evolution of species’ borders is largely unknown. There is some evidence that peripheral populations may be evolutionarily constrained [12-15], but the tendency of evolutionary biologists to study trade-offs between pairs of co-varying traits is unlikely to elucidate processes that depend on linkages between whole suites of traits [6,9,14,16] as is potentially the case for the determinants of range limits.

Evolution where multiple traits co-vary can be studied using the multivariate response equation $\Delta \bar{z}=GP^{-1}S$, where $\Delta \bar{z}$ is the vector of mean trait responses, **G** is the additive genetic variance-covariance matrix, **P** is the phenotypic variance-covariance matrix, and **S** is the vector of selection differentials [5]. Such multivariate approaches are now commonly used to understand how populations might respond to selection e.g. [7-11,17-22]. A major obstacle, however, to studying multivariate trait selection and evolution is the accurate estimation of **G**[23]. Estimating **G** requires large-scale, controlled, captive-breeding studies, or the collection of groups of relatives from the wild. Using either approach is unrealistic for all but a few species. **P**, however, may be a useful substitute for **G** in such analyses [11,21,24] where direct estimation of **G** is not feasible. While **G** and **P** are not strictly equivalent, **P** should reflect the structure of **G**, especially for traits with high heritabilities [5]. Indeed, similarity between **P** and **G** has been supported by empirical studies [23-27] but see [28]. Moreover, **P** is likely to be estimated with less error than **G,** and therefore, may represent **G** better than **G** estimated with large error [29,30]. **P** also imposes an upper limit of dimensionality on **G**[28]. Investigations of **P**, therefore, should provide some insight into the structure of **G,** and consequently, are beginning to gain greater currency in evolutionary research e.g. [11,31-35].

As selection is blind to genotype, acting solely on phenotypic variation, the ability of species to evolve will be determined first by the phenotypic variation exposed to selection. It is well established that phenotypic traits in populations of the same species can vary among geographic locations [21] and citations therein. In contrast, comparatively little is known about how phenotypic traits co-vary over similar distances, but some evidence suggests that while **P** may not remain constant, it can show substantial stability even over large distances [26,32]. Better knowledge of such stability, or otherwise, could then be used to infer selective regimes imposed on populations and species across space and how they may respond to selection in the future [27,33-36]. For example, stability of **P** between centre and range edge populations, despite an expected shift in the selective landscape from stabilizing to directional selection [1], may infer evolutionary constraint. Alternatively, re-alignment of **P**, or changes in its shape could instead indicate responses to selection, or constraint, depending on the details of any such changes in **P**. For example, the reorientation of **P** between a range edge and centre may indicate a response to selection, if such a reorientation reflects a change in the direction of selection. Conversely, if **P** has lower rank or there is greater morphological integration in range edge populations, there would be less opportunity to respond to changes in the selective regime.

Our current lack of knowledge of how **P** varies among populations and species, and what knowledge of such variation might tell us about the operation of evolutionary processes, is exemplified by the study of the evolution of species’ geographic borders. In many species, no obvious limits to dispersal exist at their current range boundaries, and despite selective advantages associated with range expansion, range margins often appear to remain stable. Populations at, or toward, the edge of a species’ geographic range, however, may experience quite different selective landscapes, and possibly greater drift, than their more centrally located counterparts [1,4]. Because of the complexity of processes that can operate on populations at range edges and the diversity of traits involved, taking a multivariate approach to the study of range edges should aid our understanding of their evolution and maintenance [4]. If so, differences in **P** between centers and edges of species ranges may be informative about how these populations could respond to selection. Further limiting our current ability to draw inferences regarding the evolution of geographic ranges has been the tendency to investigate series of populations of a particular species at varying distances from a range edge. Such designs make it difficult to separate the effects of distance to range edge from geographic location per se; interspecific comparisons and contrasts between groups of species have been suggested as an alternate and potentially more powerful approach [2]. That is, for a fixed set of resources, comparative approaches can increase the number of contrasts between centers and edges while controlling local environmental variation.

Understanding how geographically marginal populations might respond to selection is becoming increasingly urgent given the current threats of climate change, habitat fragmentation, and invasive species. With this, and the issues outlined above in mind, we adopted a comparative analytical approach that controls for geographic location and compares multiple phenotypic traits simultaneously through the analysis of **P**. Specifically, we investigated patterns of phenotypic covariance at species’ borders in reef fishes on Australia’s Great Barrier Reef (GBR). The GBR exists as a relatively contiguous habitat positioned along a predominately north–south gradient of approximately 2000 km. Many fish species inhabiting the GBR have distributions that extend well beyond both its northern and southern limits. Many others, however, have geographic range margins within, or toward the ends, of the GBR. Additionally, fish populations along the length of the GBR can be connected by gene flow over considerable distances during their larval phase, yet also exhibit detectable genetic structure over its length [37-39]. These population genetic patterns indicate that gene flow from central to marginal populations may be sufficient to disrupt local adaptation at range edges, but that gene flow is insufficient to render the local populations along the GBR effectively a single population. Across this geographic gradient there are also substantial biotic and abiotic gradients that may impose selection pressures on reef fishes of the GBR that could be important in determining their geographic range limits. For example, regional fish diversity is approximately 40% [40] and mean monthly maximum and minimum water temperatures 2 – 3°C greater (Source: data.aims.gov.au) in the north.

**Figure 1:**
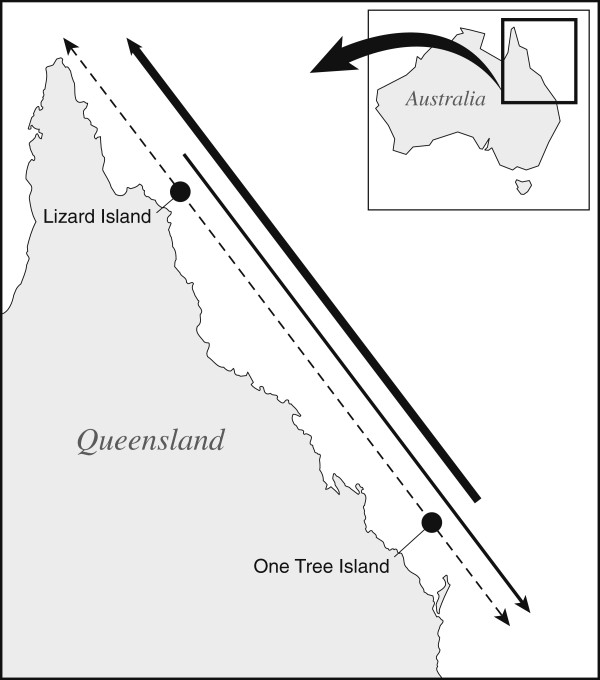
**Sampling design.** The black dots indicate the two sampling localities on the Great Barrier Reef: Lizard Island (14°40’S, 145°28’E) in the north and One Tree Island (23°25’S, 151°55’E) in the south. The thick solid arrow represents the distribution of *Amblygobius rainfordi*, and *Chrysiptera rollandi*, two species for which One Tree Island is near the southern margin of their distribution. The thin solid arrow represents the distribution of *Amphiprion akindynos*, the Lizard Island population of which is near the northern margin of this species’ distribution. The dashed line represents the distribution of the three congeneric control species whose ranges extend both north and south beyond the limits of the GBR. All species were sampled at the two locations indicated; the solid arrows are displaced for illustrative purposes.

To explore if and how the extent and pattern of phenotypic covariance changes toward the edges of geographic ranges, we estimated **P** for external morphological traits between central and marginal populations of three reef fish species sampled from three different taxonomic families (Figure 1, Tables 1, 2). We then compared these to **P** estimated for populations of congeneric species at the same sites, but for which these sites represented two geographically central locations. External morphological traits were chosen for these analyses because they could be quickly and reliably measured [32], and because morphological traits generally have higher heritabilities than life-history traits [26]. These morphological traits, therefore, should act as a better proxy for **G** than would a comparable set of life-history traits. The structure of **P** between paired populations was examined between species and within genera. We also examined pair-wise correlations of these morphological traits between sites within species, the extent of morphological integration among traits, and the ranks of **P** within sites and species to test the degree to which characteristics of **P** at geographic range edges may constrain or facilitate evolution.

**Table 1:** Range attributes of species compared: C, NB, and SB denote a population toward the centre, northern, or southern border of their geographic ranges, respectively

		**Range limit**			**Distance to border**	
**Species name**	**Population contrast**	**North**	**South**	**Range size**	**North**	**South**
		**(°lat.)**	**(° lat.)**	**N-S (° lat.)**	**(° lat.)**	**(° lat.)**
*Amblygobius phalaena*	C/C	25	−34	59	39	−11
Amblygobius rainfordi*	C/SB	9	−24	33	23	−1
*Amphiprion akindynos*	NB/C	−11	−33	22	3	−10
*Amphiprion melanopus*	C/C	20	−33	53	34	−10
*Chrysiptera rex*	C/C	24	−33	57	38	−10
*Chrysiptera rollandi*	C/SB	14	−24	37	28	−1

Distance to the northern border is estimated from Lizard Island, and the southern border from One Tree Island. (see text for data sources).

*Synonymized as *Koumansetta rainfordi* (see text for further details).

**Table 2:** Summary of ecological traits for each species studied

**Species**	**Habitat**^**++**^	**Diet**^**++**^	**PLD (days)**
*Amphiprion melanopus*	Lagoon or outer reef, commensal with anemones	Planktonic copepods and algae	11 ± 0.3(s.d.)*
*Amphiprion akindynos*	As above	As above	11.7 ± 0.3(s.d.)*
*Chrysiptera rex*	Upper reef slope	Algae	18.2 ± 0.4(s.d.)*
*Chrysiptera rollandi*	Cosmopolitan	Zooplankton and algae	16.5 ± 3.9(s.d.)*
*Amblygobius phalaena*	Lagoon or sub-tidal reef flat, burrows in sand or rubble	Small benthic invertebrates and algae	28.5 ± 1.1(s.d.)*
Amblygobius rainfordi**	Lagoon or sub-tidal ref flat	As above	30.5 ± 0.6(s.d.)*

*PLD*: pelagic larval duration.

^++^[41]; *[37,42]; **Synonymized as *Koumansetta rainfordi* (see text for further details).

## Results

**Figure 2:**
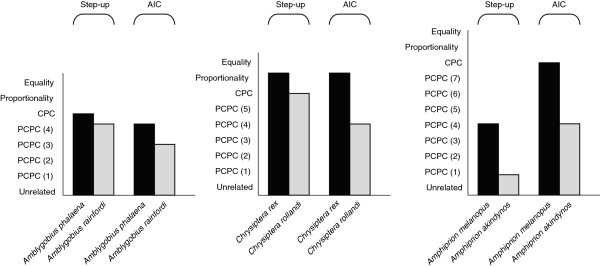
**Common principal component analyses comparing phenotypic covariance matrices from two populations (Lizard Island and One Tree Island) for six species of coral reef fishes.** The grey bars represent the three species whose populations at these locations include one geographically marginal population and one toward the edge of their distribution. The black bars represent the three congeneric control species for which the two sampled populations were located toward the center of their distribution. For each congeneric pair, the results of the step-up approach and the AIC model fitting approach are reported. On the Y-axis are different possible models of the level of similarity between two matrices as dictated by the Flury hierarchy. This hierarchical set of models begins with unrelated matrices and moves through a series of partial common principal component (PCPC) models, each with an increasing number of common components, before reaching a model where firstly all components are common (CPC), all components are common and eigenvalues are proportional (proportional), and then all axes and eigenvalues are equal (equal). The number of models compared varies based on the number of morphological traits estimated. The left hand panel depicts a comparison with a northern border. The other two panels depict comparisons with southern borders.

There was less shared phenotypic covariance structure between central and marginal populations of each species examined, than between the two centrally distributed populations of its congener at the same locations (Figure 2). In other words, the phenotypes of individuals living in populations close to range margins differed from individuals of the same species living in more centrally located populations more than would be expected from geographic separation alone. Although there was some variation in the extent of differences exhibited between central and marginal populations, the result was robust to both the analytical technique used to assess differences, and whether the marginal population represented a northern or southern border (Figure 2).

Between these two distant locations, the two populations of each of the three control species, those that do not have a range margin along the length of the GBR, typically retained all axes of phenotypic variation (eigenvectors) in common and differed only in the amount of variation associated with these axes (eigenvalues) (Figure 2). The exceptions to this were the *A. phalaena* populations which when compared using the AIC method shared all but one of their eigenvectors, and the *A. melanopus* populations whose step-up analysis indicated only 4 out of 8 axes in common. For both these species, however, the alternate CPC method still indicated a full set of common axes. In contrast to this stability, comparisons of the three species whose populations over the same distance included a geographic margin indicated that in no case had these populations retained all axes of phenotypic variation in common (See Additional file 1 for full CPC results).

In all three congeneric comparisons, there was some overlap in the 95% confidence set of models (Table 3) indicating that some caution is advisable when interpreting the strength of the differences identified here. However, in all cases, the Akaike weights indicated good support for the model selected, with the best models being 50-100% (1.5 – 2 times) more likely than the next best model (Additional file 1). Additionally, for *A. phalaena* and *A. rainfordi*, the species pair with greatest overlap in 95% CI model sets (Table 3), the next best models indicated greater stability in the central-central comparison and less stability in the central-marginal comparison. Taken together, these results suggest that, our interpretation of differences between the species sampled toward edges of their geographic ranges and their congeneric control species is supported.

**Table 3:** Nine-five percent confidence sets of models describing the similarity between the phenotypic covariance matrices from two populations for each of six reef fish species in three congeneric comparisons

***Amblygobious phalaena***	Amblygobius rainfordi*	***Amphiprion melanopus***	***Amphiprion akindynos***	***Chrysiptera rex***	***Chrysiptera rollandi***
		**CPC**			
		PCPC(7)		**Prop.**	
		PCPC(6)	PCPC(6)	CPC	CPC
CPC	CPC		PCPC(5)	PCPC(5)	PCPC(5)
**PCPC(4)**	PCPC(4)	PCPC(4)	**PCPC(4)**		**PCPC(4)**
PCPC(3)	**PCPC(3)**		PCPC(3)		PCPC(3)
PCPC(2)	PCPC(2)				
	PCPC(1)		PCPC(1)		
				Unrelated	

*Synonymized as *Koumansetta rainfordi* (see text for further details).

In each case, the species on the left hand side of the pair was sampled from two centrally distributed populations whereas the species on the right hand side contains one central and one geographically marginal population. Bolded entries correspond to the best model in that set as determined by the AIC model fitting approach.

### Pair-wise comparisons of morphological traits

The number of stronger and weaker pair-wise correlations between morphological traits within a population was unrelated to whether the comparisons were between a central and a border population, or between two central populations. The number of significant differences in trait correlations was also unrelated to a population’s status as central or edge. In contrast, 80% of correlations between traits were stronger at the northern location (Additional file 2).

### Potential for evolutionary constraint

Based on the correlatedness of traits, there was no evidence that border populations were, in general, either more or less constrained than their more centrally located counterparts. Border populations spanned the range between high, intermediate, and low values of SD(λ) compared to the other populations of their congeners (Table 4). Similarly, ranks of **P** were unrelated to geographic location, or proximity to a geographic border as all population-by-species **P**s were full rank (Table 4).

**Table 4:** Correlatedness of morphological traits and ranks of P within populations of reef fishes

**Species name**	**Population**	**No. of traits (N)**	**Sample size (M)**	**N/M**	relSD(λ)*	**Matrix rank**
*Amblygobius phalaena*	C (LZI)	6	66	0.09	0.91	6
	C (OTI)	6	69	0.09	0.85	6
Amblygobius rainfordi**	C (LZI)	6	65	0.09	0.81	6
	SB (OTI)	6	47	0.13	0.80	6
*Amphiprion akindynos*	NB (LZI)	9	53	0.17	0.89	9
	C (OTI)	9	76	0.12	0.85	9
*Amphiprion melanopus*	C (LZI)	9	47	0.19	0.87	9
	C (OTI)	9	67	0.13	0.86	9
*Chrysiptera rex*	C (LZI)	7	80	0.09	0.60	7
	C (OTI)	7	76	0.09	0.71	7
*Chrysiptera rollandi*	C (LZI)	7	56	0.13	0.76	7
	SB (OTI)	7	69	0.10	0.62	7

*Standardized for sample size.

**Synonymized as *Koumansetta rainfordi* (see text for further details).

## Discussion

Substantial differences in phenotypic covariation were observed in populations of reef fishes at both northern (warm) and southern (cool) range margins compared to populations closer to their range centers. This instability of phenotypic covariation was greater than would be expected by geographic separation alone, and is in contrast to the stability of **P** seen both in the congeneric controls used here, and in previous matrix comparisons of some additional reef fish species at these locations [32]. In the absence of a geographic margin, **P** typically retained all axes of variation in common, but differed in the amount of variation along these axes. This is suggestive of a considerable commonality of selective pressures encountered and evolutionary responses [27] by these populations. It also suggests that **E** either has little influence on **P** or has been canalized to reflect **G** in these species. The consistency of this result is good reason to believe that **P** may be informative of **G** for the populations studied here. Given the variety of life histories and morphologies represented by these species, and differences in environmental conditions experienced by these edge populations, the changes in **P** between central and marginal populations appear to be independent of these proximate factors, and are more likely the consequence of evolutionary processes operating in marginal populations.

The instability in patterns of morphological covariation in border populations was only observed in the second and lower order axes that primarily represent components of shape. Shape, however, is integral to the ecology of coral reef fishes [43], and shape in fishes can be closely tied to fitness and be selected [44]. While it is tempting to try and link the differences in structure of **P** in marginal populations as revealed by these CPC results to the adaptive nature of the measured traits, there have been numerous issues raised about the biological interpretation of these orthogonal abstractions [45].

Indeed, the greater structural differences observed here in **P** in comparisons between centrally and marginally located populations relative to their congeners in the absence of a species’ border, was not reflected in the pair-wise correlations observed between traits. More frequently, trait correlations were stronger in the north irrespective of the positioning of those populations with respect to geographic limits. A similar pattern was reported for two species of grasshopper in which stronger covariances were observed where temperatures were greater [21]. Given the observation of generally stronger correlations in the north in these fish populations, it is all the more remarkable that differences in the structure of **P** related to the position of populations relative to range edges were detected.

Differences in the structure of **P** between these central and marginal populations indicate that a unique subset of phenotypic space is accessible to selection in these marginal populations [16]. Whether selection on these phenotypes has led to their adaptive evolution, or has been constrained as a result of these differences in P, is difficult to infer with certainty in the absence of estimates of selection on these traits. Some insight, however, into whether these populations have been constrained can be gleaned from the examination of the shapes of **P**. For example, unless selection happens to be oriented in the same direction as the axis of greatest variation, phenotypic covariance distributed more unevenly among axes, or among fewer axes, should be indicative of greater constraint [20] to the degree to which **P** reflects **G**. Neither the standard deviations of eigenvalues, nor the ranks of **P**, were related to the proximity of a species’ border. Therefore, these results more likely reflect responses to processes operating at geographic range margins other than phenotypic conservatism resulting from evolutionary constraint. Nevertheless, these edge populations would be expected to respond differently to the same selection pressures experienced by their centrally located relatives.

Direct estimation of multivariate selection in these populations is beyond the scope of this study. It is difficult, therefore, to move beyond speculation as to which processes may have shaped the observed differences in phenotypic covariation. The differences in **P** seen here are of the same magnitude as those reported previously as evidence of locally distinct selection regimes [46] and even morphological divergence between species [47]. It is, however, also worth considering the effects of other processes such as differential phenotypic expression due to different environments, or demographic stochasticity, both of which could cause differences in **P** between central and marginal populations. We attempted to control the effects of environment by using a comparative analysis. It is possible, however, that selection, which may otherwise canalise the expression of environmental variation, is less effective in marginal populations as a consequence of gene swamping [48]. Similarly, although the changes in **P** between central and marginal populations were greater than those typically associated with genetic drift where proportionality is used as a criterion [49], non-proportional changes as seen here may also result from the actions of drift and cannot be rejected out-of-hand until better diagnostics are developed [27].

Associated with the predictions of small population size and directional selection, is the expectation of reduced genetic variation in marginal populations [1]. Although it is less clear how phenotypic variation will respond to the same processes, population genetics theory suggests that a decrease in phenotypic variation should also be evident [50], especially in the case of morphological traits [51]. The lack of any obvious reduction here in phenotypic variation in marginal populations that selection could act on (i.e. reduced matrix ranks or greater trait integration estimated by SD(λ)), however, is not necessarily surprising. The loss of genetic variation in traits could be offset by increased expression of environmental variation in the phenotype. Alternatively, fluctuations in the direction of selection, quite possible in marginal populations exposed to changes in population densities [52], could increase phenotypic variation [53]. As a result, it remains unclear exactly how the extent of phenotypic variation should evolve at range margins.

Irrespective of the exact processes contributing to this result, we have demonstrated that geographical marginality can be associated with substantial change in **P**. More work, both theoretical and empirical, is needed to assess the generality of this result in other taxa, and if it proves to be a common pattern, its causes and consequences for evolutionary rates and directions. In the meantime, it appears likely that for reef fishes at least, differences in **P**, and possibly **G**, can result from conditions encountered by wild populations living close to their range edges. As patterns of phenotypic variation can have a substantial influence over a population’s evolutionary trajectory [18,54], such differences in phenotypic covariance are likely to have important consequences for evolution at range margins.

Given contemporary threats posed by climate change, invasive species, and landscape fragmentation, understanding the forces shaping species’ ranges is of critical importance to the long-term conservation of biodiversity. Attempts to predict future range migrations, or the longer-term persistence of populations in new locations, or with changing environmental conditions, while ignoring changes in phenotypic covariance at range edges may seriously undermine our predictions of the abilities of species to adapt, and thereby, our estimates of extinction risk. Acquiring a greater understanding of evolutionary potential in marginal populations should be accorded very high priority by evolutionary and conservation biologists alike.

## Conclusions

Phenotypic covariation was greater between populations of reef fishes at both northern (warm) and southern (cool) range margins compared to populations closer to their range centers. Given the variety of life histories and morphologies represented by these species, and differences in environmental conditions experienced by these edge populations, the changes in **P** between central and marginal populations appear to be independent of these proximate factors, and are more likely the consequence of evolutionary processes operating in marginal populations. Neither the degree of morphological integration of the traits studied, nor the ranks of **P**, indicated greater evolutionary constraint at range edges. Instead, these differences in **P** may reflect structural change in **P** caused by selection or drift, and the potential to evolve in the future.

## Methods

### Sampling design

We studied *Amblygobius rainfordi* (Gobiidae) (since synonymised as *Koumansetta rainfordi*, a sister genus in the subfamily Gobiinea), *Amphiprion akindynos* (Pomacentridae), and *Chrysiptera rollandi* (Pomacentridae). Specimens of these species were collected in the vicinity of two locations, approximately 1200 km apart, on the GBR: Lizard Island (14°40’S, 145°28’E) in the north, and One Tree Island (23°25’S, 151°55’E) in the south (Figure 1). For two of these species, *A. rainfordi* and *C. rollandi*, the One Tree Island populations were within 50 kilometres of their southern distributional limit, whereas, both have been recorded a few thousand kilometres north of Lizard Island. *A. akindynos,* on the other hand, is regularly found over 1000 kilometres south of One Tree Island, but is rarely recorded more than a few hundred kilometres north of Lizard Island. These geographic distributions were estimated from publicly available databases (e.g. The Altas of Living Australia (http://www.ala.org.au), The Ocean Biogeographic Information System (http://www.iobis.org)) and are summarized in Table 1.

Theory predicts that populations across much of a species’ range may not be optimally adapted as a result of gene flow from the center to the periphery of ranges [55]. Therefore, we are confident that the populations sampled here are sufficiently close to their range limits to sample the environmental conditions of their range margins. For our purposes here then, Lizard Island represents a sampling location for the northern border of *A. akindynos*, whereas One Tree Island represents a sampling location for the southern border of the other two species. These three species also encompass a diverse range of morphologies and life histories, including pelagic larval durations (PLD) that vary between 10 and 31 days [37,42] (Table 2), and were chosen to maximize the generality of any patterns that emerged.

The morphology of ectotherms is sensitive to environmental conditions, particularly temperature [56], which differs between the northern and southern sampling locations used here. Also, as **P** is a function of **G** and **E**, where **E** is the matrix of environmental covariation [57], it is necessary to separate, as much as possible, the effects of environment from the effects of geographic marginality. To control the central/marginal comparisons for changes in **P** associated with environmental differences experienced by these fishes at different locations, and to provide a basis to compare changes in **P** across a geographic range in the absence of a geographic border, we adopted a comparative approach. For each of the species we examined that exhibits a range edge along the GBR, a congeneric species with similar life histories (Table 2), but whose distribution extends well beyond the northern and southern extremities of the GBR were included in this comparative analysis (Figure 1). The three congeneric species used were *Amblygobius phalaena*, *Amphiprion melanopus*, and *Chrysiptera rex*. All of these species are found a minimum of 10° latitude both north and south of the collection locations used here. Under this sampling design, differences in **P** between central and marginal populations compared to those seen between the populations of their paired species can be interpreted as indicative of processes operating in geographically marginal populations.

### Trait estimation

Specimens were collected and analysed using the methods described by Game and Caley [32]. A total of 1040 fish were sampled including 113 *A. rainfordi*, 135 *A. phalaena*, 129 *A. akindynos*, 114 *A. melanopus*, 124 *C. rollandi*, and 156 *C. rex*.

In order to incorporate phenotypic variation among traits across different ontogenetic stages, individuals across the entire post-settlement size range of each species were sampled. Nine morphological traits: minimum caudal peduncle depth (CPD), dorsal fin length (DFL), nape length (NPL), head length (HDL), eye diameter (EYD), body depth at the back of the head (BDH), pectoral fin depth (PFD), inter pelvic/anal fin distance (IPA), and anal fin length (AFL), were estimated to the nearest 0.1 mm for each of the individuals used in this study. These nine traits were chosen to represent major morphological structures common to all these species, were distributed relatively evenly over the surface of each fish, and didn’t traverse multiple structures that may not co-vary isometrically. Two traits (i.e., NPL and HDL) were removed from the analysis of the *Chrysiptera* populations and three traits (i.e., HDL, EYD and PFD) from the analysis of *Amblygobius* populations because they could not be measured accurately (see [32] for further details). All traits used here were estimated by the same individual (EG) to eliminate inter-observer variation and % measurement error for all traits retained for analysis was estimated to be < 1% [32]. Using these estimates of phenotypic traits, phenotypic variance-covariance matrices (**P**) were calculated for each population.

### Matrix comparisons

Covariance matrices can share a series of relationships between the extremes of equality and inequality [58]. To determine the level of structural similarity between matrices, Phillips’ CPCA software [59] was used. This common principal component (CPC) analysis was run using both the step-up method, which fits within a traditional hypothesis testing framework, and the Akaike Information Criterion (AIC) method, which selects the best fitting model given the information available. Because AIC estimates the distance between two models, it is also possible to calculate a weight (*w*_*i*_) for each model, interpreted as the probability that model *i* is the actual best model given the available set of models [60]. Using these Akaike weights ninety-five percent confidence limits were estimated, in effect producing a 95% confidence set of models. This set of models will include the actual best model in 95% of cases and should help in interpreting the results in the context of the sampling variation likely for the sample sizes used here [47]. For a more complete discussion of the techniques used here see Game and Caley [32].

### Sources of structural differences between P matrices

The relative strengths of all pair-wise trait correlations of each species were compared between One Tree Island and Lizard Island. To do so, we conducted a Bayesian analysis where the traits for each species were observations from a multivariate Gaussian distribution. The prior for the mean vector and variance-covariance matrix was the conjugate normal-inverse-Wishart prior [61]. A Markov chain Monte Carlo (MCMC) algorithm was implemented in Matlab to estimate the model. The advantage of using this Bayesian model is the MCMC output allows straightforward estimation of the probability distribution of the difference between the correlation of traits *i* and *j* at One Tree Island and Lizard Island. Mathematically, if r_o_(t_*i*_, t_*j*_|Data) is the posterior distribution of the correlation between trait i and trait j at One Tree Island and r_l_ (t_*i*_, t_*j*_|Data) is the posterior distribution of the correlation between trait *i* and trait *j* at

$$p\left(r_{\mathrm{ij}}\left|\mathrm{Data}\right)\right)=p\left(r_{o}\left(t_{i},t_{i}\right)-r_{1}\left(t_{i},t_{i}\right)\left|\mathrm{Data}\right)\right)$$

where p(r_*ij*_ | Data) is the posterior distribution of the difference in the correlation between One Tree Island and Lizard Island. If a particular trait correlation was greater/lesser at One Tree Island than at Lizard Island, p(r_*ij*_ | Data) will be mainly greater/less than zero. Differences in trait correlations were considered significant if zero was contained in the 1st or 99th percentile of p(r_*ij*_ | Data).

### Potential for evolutionary constraint

The potential for evolutionary constraint given observed **P**s was examined two ways. First, the degree of morphological integration of traits in each population was examined by estimating the standard deviation of the eigenvalues (SD(λ)) for each **P**, standardized for the number a traits measured and sample size [62]. SD(λ) estimates the degree of co-variation among all traits represented in an estimate of **P**. Where SD(λ) is greater, changes in **P** should be relatively more constrained. We compared the SD(λ)s estimated for central populations with the SD(λ)s estimated for the border populations.

Ranks of **P** were also estimated and compared. The rank of a matrix equals its number of non-zero eigenvalues and describes its dimensionality. The lower the rank, the fewer the axes along which variation is distributed. Consequently, the lower the rank of **P**, the more constrained the response to selection in multi-trait space. Because **P** defines the upper bound of **G**, constraint imposed by **G** may be greater than that imposed by **P** e.g. [28]. Therefore, **P** provides a conservative estimate of constraint.

Methods for estimating ranks of **P** have been developed based on the repeated measurement of individuals e.g. [28,63]. Repeated measurements of the fish studied here were unavailable. Previously, however, we estimated percentage measurement error for these traits [32]. We used these estimates of measurement error to simulate repeated measures using the method and code reported in Pavlicev et al. [63]. For each estimated individual-by-trait combination, we simulated two, repeated measurements by adding and subtracting the percentage measurement error to and from the observed value, respectively.

## Competing interests

The authors declare that they have no competing interests.

## Authors’ contributions

MJC and ETG conceived the study, ETG collected the data, MJC, EC and ETG analyzed the data and wrote the paper. All authors read and approved the final manuscript.

## Supplementary Material

Additional file 1 **Common principal component analyses comparing two populations (Lizard Island and One Tree Island) for three species pairs of coral reef fish species.** Up to 11 possible levels of similarity between variance-covariance matrices are listed in descending order of similarity. The number of levels of similarity was determined by the number of traits measured. *P* values are reported for the step-up procedure, as are Akaike’s Information Criterion and Akaike weights. The bolded entries correspond to the model of matrix similarity selected by each procedure. See text for further detail regarding the model selection procedures used. Click here for file

Additional file 2 **The relative strengths of pair-wise correlations between morphological traits for each species compared between One Tree Island and Lizard Island.** A particular trait correlation greater at One Tree Island is indicated by a positive value and a negative value if greater at Lizard Island. Differences in trait correlations were considered significant if zero was contained in the 1^st^ or 99^th^ percentile of the posterior distribution and are indicated in bold type. Click here for file
