# Supplementary material for: Phenotypic covariance at species’ borders
Source: BMC Evol Biol. 2013 May 28;13:105. doi: 10.1186/1471-2148-13-105 (PMC3681583; doi:10.1186/1471-2148-13-105)
Supplement: Additional file 1 — Common principal component analyses comparing two populations (Lizard Island and One Tree Island) for three species pairs of coral reef fish species. Up to 11 possible levels of similarity between variance-covariance matrices are listed in descending order of similarity. The number of levels of similarity was determined by the number of traits measured. P values are reported for the step-up procedure, as are Akaike’s Information Criterion and Akaike weights. The bolded entries correspond to the model of matrix similarity selected by each procedure. See text for further detail regarding the model selection procedures used. [file 1471-2148-13-105-S1.docx]

**Additional file 1 - Common principal component analyses comparing two populations (Lizard Island and One Tree Island) for three species pairs of coral reef fish species.** Up to 11 possible levels of similarity between variance-covariance matrices are listed in descending order of similarity. The number of levels of similarity was determined by the number of traits measured. *P* values are reported for the step-up procedure, as are Akaike’s Information Criterion and Akaike weights. The bolded entries correspond to the model of matrix similarity selected by each procedure. See text for further detail regarding the model selection procedures used.

| Model | | Step-up (*P*) | | AIC | | Akaike weight |  |
| --- | --- | --- | --- | --- | --- | --- | --- |
| *Amblygobius phalaena* | |  | |  | |  |  |
| Equality | | 0.0014 | | 72.378 | | 0.001 |  |
| Proportionality | | 0.0000 | | 64.133 | | 0.001 |  |
| CPC | | **0.0558** | | 34.633 | | 0.226 |  |
| PCPC(4) | | 0.4296 | | **32.974** | | **0.517** |  |
| PCPC(3) | | 0.2461 | | 35.284 | | 0.163 |  |
| PCPC(2) | | 0.1994 | | 37.139 | | 0.065 |  |
| PCPC(1) | | 0.2103 | | 39.142 | | 0.024 |  |
| Unrelated | |  | | 42.000 | | 0.006 |  |
|  | |  | |  | |  |  |
| *Amblygobius rainfordi* | |  | |  | |  |  |
| Equality | | 0.7573 | | 38.137 | | 0.024 |  |
| Proportionality | | 0.0159 | | 40.042 | | 0.009 |  |
| CPC | | 0.0665 | | 36.090 | | 0.067 |  |
| PCPC(4) | | 0.0418 | | 34.724 | | 0.133 |  |
| PCPC(3) | | **0.1561** | | **32.374** | | **0.432** |  |
| PCPC(2) | | 0.4166 | | 33.150 | | 0.293 |  |
| PCPC(1) | | 0.3888 | | 37.227 | | 0.038 |  |
| Unrelated | |  | | 42.000 | | 0.004 |  |
| *Amphiprion melanopus* | |  | |  | |  |  |
| Equality | | 0.0003 | | 110.721 | | 0.001 |  |
| Proportionality | | 0.0000 | | 99.800 | | 0.001 |  |
| CPC | | 0.3476 | | **73.113** | | **0.388** |  |
| PCPC(7) | | 0.1269 | | 74.231 | | 0.222 |  |
| PCPC(6) | | 0.6991 | | 74.102 | | 0.236 |  |
| PCPC(5) | | 0.0229 | | 78.674 | | 0.024 |  |
| PCPC(4) | | **0.9045** | | 75.328 | | 0.128 |  |
| PCPC(3) | | 0.2168 | | 83.755 | | 0.002 |  |
| PCPC(2) | | 0.0711 | | 87.453 | | 0.001 |  |
| PCPC(1) | | 0.0717 | | 88.410 | | 0.001 |  |
| Unrelated | |  | | 90.000 | | 0.001 |  |
|  | |  | |  | |  |  |
| *Amphiprion akindynos* |  | |  | |  | | |
| Equality | 0.8083 | | 120.282 | | 0.001 | | |
| Proportionality | 0.0000 | | 122.223 | | 0.001 | | |
| CPC | 0.0838 | | 87.636 | | 0.012 | | |
| PCPC(7) | 0.0905 | | 86.647 | | 0.019 | | |
| PCPC(6) | 0.0157 | | 85.841 | | 0.029 | | |
| PCPC(5) | 0.0501 | | 81.469 | | 0.259 | | |
| PCPC(4) | 0.5179 | | **79.986** | | **0.544** | | |
| PCPC(3) | 0.1033 | | 85.764 | | 0.030 | | |
| PCPC(2) | 0.0140 | | 87.212 | | 0.015 | | |
| PCPC(1) | **0.2923** | | 83.625 | | 0.088 | | |
| Unrelated |  | | 90.000 | | 0.004 | | |
|  | |  | |  | |  |  |
| *Chrysiptera rex* | |  | |  | |  |  |
| Equality | | 0.0008 | | 64.671 | | 0.005 |  |
| Proportionality | | **0.2575** | | **55.332** | | **0.514** |  |
| CPC | | 0.8659 | | 59.588 | | 0.061 |  |
| PCPC(5) | | 0.2150 | | 61.560 | | 0.023 |  |
| PCPC(4) | | 0.2663 | | 62.485 | | 0.014 |  |
| PCPC(3) | | 0.0745 | | 64.530 | | 0.005 |  |
| PCPC(2) | | 0.1150 | | 64.018 | | 0.007 |  |
| PCPC(1) | | 0.1017 | | 65.162 | | 0.004 |  |
| Unrelated | |  | | 56.000 | | 0.368 |  |
| *Chrysiptera rollandi* |  | |  | |  | | |
| Equality | 0.0000 | | 63.889 | | 0.001 | | |
| **Proportionality** | 0.0169 | | 38.667 | | 0.044 | | |
| CPC | **0.1423** | | 35.205 | | 0.248 | | |
| PCPC(5) | 0.1086 | | 35.051 | | 0.268 | | |
| PCPC(4) | 0.3171 | | **34.611** | | **0.334** | | |
| PCPC(3) | 0.5535 | | 37.083 | | 0.097 | | |
| PCPC(2) | 0.5699 | | 42.058 | | 0.008 | | |
| PCPC(1) | 0.6498 | | 48.199 | | 0.001 | | |
| *Unrelated* |  | | 56.000 | | 0.001 | | |
|  | |  | |  | |  |  |
