# Supplementary material for: Phenotypic covariance at species’ borders
Source: BMC Evol Biol. 2013 May 28;13:105. doi: 10.1186/1471-2148-13-105 (PMC3681583; doi:10.1186/1471-2148-13-105)
Supplement: Additional file 2 — The relative strengths of pair-wise correlations between morphological traits for each species compared between One Tree Island and Lizard Island. A particular trait correlation greater at One Tree Island is indicated by a positive value and a negative value if greater at Lizard Island. Differences in trait correlations were considered significant if zero was contained in the 1st or 99th percentile of the posterior distribution and are indicated in bold type. [file 1471-2148-13-105-S2.docx]

**Additional file 2 - The relative strengths of pair-wise correlations between morphological traits for each species compared between One Tree Island and Lizard Island.** A particular trait correlation greater at One Tree Island is indicated by a positive value and a negative value if greater at Lizard Island. Differences in trait correlations were considered significant if zero was contained in the 1^st^ or 99^th^ percentile of the posterior distribution and are indicated in bold type.

| *Amblygobius phalaena* (C/C) | | | |  | |  | |  | |  | |
| --- | --- | --- | --- | --- | --- | --- | --- | --- | --- | --- | --- |
|  | CPD | DFL | NPL | | BDH | | IPA | | AFL | |  |
| CPD | 0 |  |  | |  | |  | |  | |  |
| DFL | -0.04 | 0 |  | |  | |  | |  | |  |
| NPL | -0.05 | -0.05 | 0 | |  | |  | |  | |  |
| BDH | -0.04 | -0.04 | -0.04 | | 0 | |  | |  | |  |
| IPA | -0.06 | -0.07 | **-0.10** | | -0.09 | | 0 | |  | |  |
| AFL | -0.04 | -0.04 | -0.06 | | -0.06 | | -0.07 | | 0 | |  |
|  |  |  |  | |  | |  | |  | |  |
| *Amblygobius rainfordi* (C/SB) | | | | |  | |  | |  | |  |
|  | CPD | DFL | NPL | | BDH | | IPA | | AFL | |  |
| CPD | 0 |  |  | |  | |  | |  | |  |
| DFL | -0.01 | 0 |  | |  | |  | |  | |  |
| NPL | -0.04 | 0.01 | 0 | |  | |  | |  | |  |
| BDH | -0.03 | 0.01 | -0.00 | | 0 | |  | |  | |  |
| IPA | -0.06 | -0.01 | -0.01 | | -0.03 | | 0 | |  | |  |
| AFL | 0.03 | 0.04 | 0.03 | | 0.02 | | 0.00 | | 0 | |  |

| *Amphiprion akindynos* (NB/C) | | | |  |  |  |  |  |  |
| --- | --- | --- | --- | --- | --- | --- | --- | --- | --- |
|  | CPD | DFL | NPL | HDL | EYD | BDH | PFD | IPA | AFL |
| CPD | 0 |  |  |  |  |  |  |  |  |
| DFL | -0.01 | 0 |  |  |  |  |  |  |  |
| NPL | -0.01 | -0.01 | 0 |  |  |  |  |  |  |
| HDL | -0.02 | -0.03 | -0.04 | 0 |  |  |  |  |  |
| EYD | -0.02 | -0.02 | -0.01 | -0.04 | 0 |  |  |  |  |
| BDH | -0.00 | -0.01 | -0.01 | -0.02 | -0.04 | 0 |  |  |  |
| PFD | -0.03 | -0.04 | -0.04 | -0.08 | -0.05 | -0.04 | 0 |  |  |
| IPA | **-0.14** | **-0.13** | **-0.12** | **-0.22** | **-0.16** | **-0.13** | **-0.14** | 0 |  |
| AFL | -0.04 | -0.02 | -0.02 | -0.03 | -0.01 | -0.03 | -0.06 | **-0.20** | 0 |
|  |  |  |  |  |  |  |  |  |  |
| *Amphiprion meleanopus* (C/C) | | |  |  |  |  |  |  |  |
|  | CPD | DFL | NPL | HDL | EYD | BDH | PFD | IPA | AFL |
| CPD | 0 |  |  |  |  |  |  |  |  |
| DFL | -0.01 | 0 |  |  |  |  |  |  |  |
| NPL | -0.01 | -0.01 | 0 |  |  |  |  |  |  |
| HDL | -0.04 | -0.04 | -0.05 | 0 |  |  |  |  |  |
| EYD | -0.06 | -0.06 | -0.05 | -0.03 | 0 |  |  |  |  |
| BDH | -0.01 | -0.01 | -0.01 | -0.04 | -0.07 | 0 |  |  |  |
| PFD | -0.03 | -0.04 | -0.05 | -0.05 | -0.05 | -0.03 | 0 |  |  |
| IPA | 0.00 | 0.00 | -0.01 | -0.09 | -0.09 | -0.01 | -0.07 | 0 |  |
| AFL | -0.02 | -0.00 | -0.01 | -0.05 | -0.06 | -0.01 | -0.05 | 0.05 | 0 |
|  |  |  |  |  |  |  |  |  |  |

| *Chrysiptera rex* (C/C) | |  |  |  |  |  |  |  |  |
| --- | --- | --- | --- | --- | --- | --- | --- | --- | --- |
|  | CPD | DFL | EYD | BDH | PFD | IPA | AFL |  |  |
| CPD | 0 |  |  |  |  |  |  |  |  |
| DFL | 0.00 | 0 |  |  |  |  |  |  |  |
| EYD | **0.27** | **0.28** | 0 |  |  |  |  |  |  |
| BDH | 0.01 | 0.01 | **0.25** | 0 |  |  |  |  |  |
| PFD | 0.05 | 0.01 | **0.21** | 0.06 | 0 |  |  |  |  |
| IPA | **0.24** | **0.23** | **0.37** | **0.27** | **0.19** | 0 |  |  |  |
| AFL | -0.06 | -0.03 | **0.23** | -0.03 | 0.03 | **0.17** | 0 |  |  |
|  |  |  |  |  |  |  |  |  |  |
|  |  |  |  |  |  |  |  |  |  |
| *Chrysiptera rollandi (C/SB)* | | |  |  |  |  |  |  |  |
|  | CPD | DFL | EYD | BDH | PFD | IPA | AFL |  |  |
| CPD | 0 |  |  |  |  |  |  |  |  |
| DFL | -0.03 | 0 |  |  |  |  |  |  |  |
| EYD | **-0.33** | **-0.28** | 0 |  |  |  |  |  |  |
| BDH | -0.03 | -0.03 | **-0.25** | 0 |  |  |  |  |  |
| PFD | **-0.15** | **-0.17** | **-0.28** | **-0.18** | 0 |  |  |  |  |
| IPA | **-0.19** | **-0.13** | **-0.23** | **-0.14** | **-0.27** | 0 |  |  |  |
| AFL | -0.03 | -0.04 | **-0.30** | -0.06 | -0.06 | -0.09 | 0 |  |  |
|  |  |  |  |  |  |  |  |  |  |
